# An Approach to Differential Diagnosis of Antiphospholipid Antibody Syndrome and Related Conditions

**DOI:** 10.1155/2014/341342

**Published:** 2014-10-14

**Authors:** Giacomo Emmi, Elena Silvestri, Danilo Squatrito, Lucia Ciucciarelli, Anna Maria Cameli, Gentian Denas, Mario Milco D'Elios, Vittorio Pengo, Lorenzo Emmi, Domenico Prisco

**Affiliations:** ^1^Department of Experimental and Clinical Medicine, University of Florence, 50134 Florence, Italy; ^2^Department of Cardiac Thoracic and Vascular Sciences, University of Padua, 35128 Padua, Italy; ^3^SOD Patologia Medica, Center for Autoimmune Systemic Diseases, Behçet Center and Lupus Clinic, AOU Careggi Hospital, 50134 Florence, Italy

## Abstract

The antiphospholipid antibody syndrome is a systemic, acquired, immune-mediated disorder characterized by episodes of venous, arterial, or microcirculation thrombosis and/or pregnancy abnormalities, associated with the persistent presence of autoantibodies, confirmed at least in two occasions 12 weeks apart, directed to molecular complexes consisting of phospholipids and proteins. Antiphospholipid antibody syndrome should always be considered as a potential diagnosis especially for young patients presenting with a history of thrombotic events, in particular when they occur without any obvious external trigger or any inherited thrombophilic mutation (even if 2006 criteria do not exclude antiphospholipid antibody syndrome in patients with other inherited or acquired prothrombotic conditions), or for women with recurrent pregnancy losses or later fetal deaths. Many other disorders are able to mimic antiphospholipid antibody syndrome, so a broad range of alternative diagnoses should be investigated and ruled out during clinical workup.

## 1. Introduction

Antiphospholipid antibody syndrome (APS) is a systemic, autoimmune, acquired disorder characterized by venous and/or arterial thrombosis and/or pregnancy morbidities, associated with the positivity, confirmed at least in two occasions 12 weeks apart, of such autoantibodies directed toward molecular complexes of phospholipids and proteins. A broad range of different diagnoses should be investigated and ruled out during diagnostic workup, because many other disorders are able to mimic APS. This entity should always be considered especially in young patients with history of thrombotic events without inherited thrombophilic mutation or external trigger or in women with recurrent pregnancy losses or later fetal deaths. According to 2006 Miyakis classification criteria, the presence of other inherited or acquired prothrombotic conditions does not exclude APS diagnosis. On the other hand, the identification of patients with the so-called APS “noncriteria” is important because these patients have often an autoimmune disorder that can evolve into a true APS during followup [1, 2].

In clinical practice, according to these classification criteria, it is possible to identify the following situations.

- *Patients with usual clinical manifestations of APS associated with positivity of antiphospholipid antibodies (aPL)*: thrombotic events in typical districts such as deep vein thrombosis of the lower limbs, pulmonary embolism, myocardial infarction, and stroke or typical pregnancy disorders. This is the typical APS.
- *Patients with unusual clinical presentation of typical manifestations of APS associated with positivity of aPL*: for example, thrombotic events in atypical districts, particularly liver, renal, adrenal, and mesenteric vessels or cerebral venous sinuses with unclear presentation and difficult diagnosis.
- *Female patients with incomplete clinical manifestations of obstetric APS associated with positivity of aPL*: pregnancy disorders not completely fulfilling Miyakis criteria (e.g., 2 consecutive abortions before the 10th week of gestation or 3 or more nonconsecutive abortions before the 10th week).
- *Patients with noncriteria clinical manifestations of APS associated with positivity of aPL*: for example, nonthrombotic pulmonary or cardiac involvement, ocular, neurological, osteoarticular, and hematological manifestations.

In (a) situation, diagnosis is usually simple. However, special attention deserves the exclusion of certain conditions, such as microangiopathic syndromes or systemic lupus erythematosus (SLE).

In (b) condition, the absence of nonspecific clinical manifestations (e.g., an acute abdominal pain due to visceral thrombosis) could make APS diagnosis more complex.

In (c) and (d) cases the patient who presents noncriterial, but suggestive, clinical features deserves a careful followup to detect early “clinical criteria” and to establish the best treatment.

Particular attention must be paid to those forms characterized by typical thrombotic events or pregnancy disorders in the absence of other causes, without aPL positivity (the so-called “seronegative APS”); probably, at the present time laboratory tests are inadequate to detect all APS patients since recently the existence of “noncriterial” potentially diagnostic antibodies has been proposed [3].

**Figure 1:**
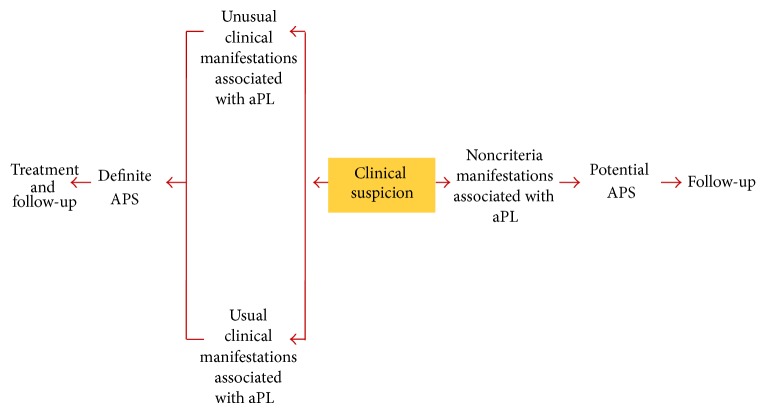
Clinical suspicion for definite and noncriterial APS.

For diagnostic algorithm, see Figure 1.

## 2. Definite APS with Usual Clinical Manifestations

Thrombotic events in APS can involve both arterial and venous vessels of any size and district, sometimes requiring a broad number of medical conditions to be ruled out (see Table 1).

### 2.1. Microangiopathic Syndromes

Thrombotic microangiopathic syndromes include thrombotic thrombocytopenic purpura (TTP), hemolytic uremic syndrome (HUS), and hemolysis, elevated liver enzymes, and low platelets (HELLP) syndrome; these disorders are characterized by platelet consumption, intravascular hemolysis with schistocytes due to red cell fragmentation, and clinical/laboratory findings of organ dysfunction; histologically they are all marked by small vessel occlusion with hyaline thrombi and fibrin deposition. Interestingly, aPL have not been rarely detected in these conditions and on the other hand APS does not infrequently show some degree of microangiopathic involvement, especially in its catastrophic form; indeed, catastrophic antiphospholipid syndrome (CAPS) is considered the most devastating manifestation of APS, fatal up to 50% of cases and characterized by multiple organ dysfunction developing in a few days, due to intravascular microthrombosis. For all these conditions the term MAPS (microangiopathic antiphospholipid syndrome) has been proposed [4, 5].

#### 2.1.1. Thrombotic Thrombocytopenic Purpura

Moschowitz syndrome, the eponym of TTP, is mainly characterized by fever, microangiopathic hemolytic anemia, thrombocytopenia, and severe neurological involvement, while renal involvement is quite rare; diagnostic markers are considered the presence of high levels of circulating ultralarge von Willebrand factor (ULVWF) multimers and schistocytes. Of note, in acquired autoimmune form, the presence of antibodies against the metalloproteinase ADAMTS13 could be useful for differential diagnosis; on the other hand aPL have been occasionally reported in TTP patients [6].

Interestingly in TTP 90% of deaths are from myocardial infarction (due to platelet thrombi in the coronary circulation). Myocardial infarction is also a frequent manifestation of arterial thrombosis in APS.

In clinical practice APS rarely presents a severe thrombocytopenia such as in TTP.

#### 2.1.2. Hemolytic Uremic Syndrome

HUS is clinically characterized by microvascular thrombosis with consequent tissue ischemia and necrosis with renal failure, thrombocytopenia, and microangiopathic anemia. There are two distinct forms: (a) diarrhea-associated HUS, typically correlated with Shiga-like toxin-producing bacteria (STEC-HUS), and (b) atypical HUS (diarrhea nonassociated) which represents a frequent form of HUS in adults [7, 8].

Clinical issues for differential diagnosis are mainly represented by the rapid onset of renal involvement and the presence of microangiopathic anemia with thrombocytopenia associated with episodes of diarrhea. aPL are usually not found in this condition.

#### 2.1.3. Hemolysis, Elevated Liver Enzymes, and Low Platelets

HELLP syndrome is more common in multiparous women between the 27th and the 37th week of gestation; clinically, it is characterized by gastrointestinal involvement (abdominal pain with nausea and vomiting), generalized edema, signs of hemolysis, elevated liver enzymes, severe thrombocytopenia, and signs of renal failure. Of note severe hypertension is not usually found in HELLP syndrome, helping to distinguish it from preeclampsia (even if they can coexist in about 70–80% of cases) [9].

Differential diagnosis is complicated by the evidence that also APS patients can experience HELLP syndrome during pregnancy [10].

### 2.2. Heparin Induced Thrombocytopenia

Another clinical condition, potentially very severe, characterized by thrombocytopenia and multiple thrombotic occlusions of small vessels, is heparin induced thrombocytopenia (HIT), which should be suspected early during heparin treatment [11].

Two major forms have been described:

- type I HIT, with favorable prognosis, occurs in approximately 10% of patients undergoing both low molecular weight heparin (LMWH) and unfractionated heparin (UH) therapy. It occurs very early usually within the first four/five days of therapy (thought to be caused by direct platelet activation from heparin) and resolves without treatment and without any complication since it is not associated with thrombotic events;
- type II HIT, which involved 1–5% of patients undergoing heparin therapy (more frequently using unfractionated heparin compared to LMWH), is associated with microthrombosis involving multiple districts affecting both the arterial and the venous vessels (in such cases the risk of mortality is estimated to be 20–30%). Diagnostic marker is considered a platelet count which falls by about 50% compared to baseline after 5–15 days from the beginning of heparin therapy. Of note in type II HIT the production of specific antiplatelet antibodies (i) may not be associated with thrombocytopenia or (ii) may be associated with thrombocytopenia but not with thrombotic complications or (iii) may be associated with both thrombocytopenia and thrombosis.

The pathogenesis of HIT has been recently better defined: during the early phase heparin binds to platelet factor 4 (PF4), generating a complex (heparin-PF4) toward which IgG antibodies are produced and this complex is also able to activate platelets via Fc*γ*RIIa receptor, causing microthrombosis and thrombocytopenia.

The detection of antibodies to heparin-PF4 complex for HIT laboratory diagnosis can be performed using either a serotonin release assay (SRA), with low sensitivity and high specificity for HIT, or an ELISA test, more sensitive but less specific for HIT. Of note the diagnosis not only is based on these tests but also is currently based on probability according to a compatible clinical scenario [12].

HIT treatment requires immediate discontinuation of heparin and the beginning of an alternative anticoagulation strategy with direct thrombin inhibitor, heparinoids or pentasaccharides.

Both APS and HIT can present thrombocytopenia (usually more severe in HIT) and thrombosis, but anamnestic clues (i.e., previous heparin exposure and the correlation with thrombocytopenia onset) and the absence of aPL could help in diagnostic workup.

### 2.3. Disseminated Intravascular Coagulation

Disseminated intravascular coagulation (DIC) is characterized by microvascular thrombosis, possibly leading to consumptive coagulopathy and bleeding, which can in turn lead to multiple organ failure; it is secondary to surgery, chronic inflammatory diseases or malignancies, and sepsis. Laboratory markers of DIC are thrombocytopenia, prolonged clotting times, increased levels of fibrin degradation products, and reduced plasma fibrinogen.

Differential diagnosis with APS could be difficult, because DIC is able to both mimic and complicate APS, especially in its catastrophic form (CAPS), which is characterized by disseminated intravascular thrombosis resulting in multiorgan failure; an aid in clinical practice could be the evidence that more frequently DIC presents thrombotic and hemorrhagic complications at the same time [13].

However, one should keep in mind that the main clinical presentation in DIC is hemorrhage and sepsis is the most common cause.

**Figure 2:**
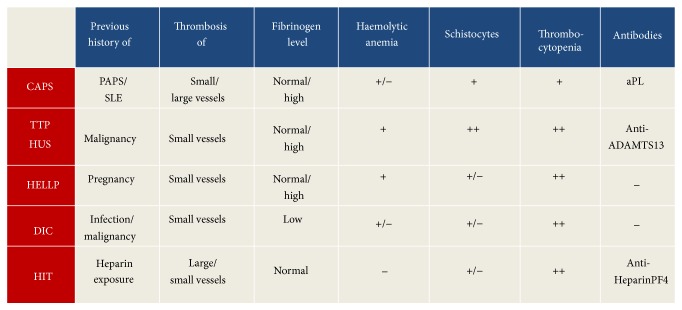
Clinical and laboratory findings in microangiopathic syndromes, DIC and HIT.

For clinical and laboratory findings in microangiopathic syndromes, DIC and HIT, see Figure 2.

### 2.4. Systemic Lupus Erythematosus

In Miyakis criteria the term “secondary APS” is considered inappropriate because SLE and APS may represent a different spectrum of the same disease, underlying the strong relation existing between these conditions [1].

Indeed not only SLE patients could present aPL positivity associated with vascular involvement or obstetrical disease, but also APS patients could present some SLE features, such as autoimmune hemolytic anemia and mild reduction in complement, anti-nuclear antibodies (ANA); interestingly, these patients can be considered as APS patients who likely will evolve into SLE over time.

Renal involvement in APS patients is not infrequent; markers to distinguish SLE renal involvement from APS ones are a significant titer of circulating ANA, the presence of other autoantibody specificities such as anti-native DNA, anti-Sm or anti-C1q, complement consumption, and specific histological findings from renal biopsy.

Given that both APS and SLE could present similar neurological symptoms and MR findings, another clinical challenge is the differential diagnosis with Neuro-SLE [14]. This discrimination is fundamental in clinical practice since in the case of APS anticoagulants are the main treatment, while Neuro-SLE requires the use of pulse high dose steroids associated with immunosuppressive drugs [15]. The detection of a significant titer of specific circulating autoantibodies such as anti-ribosomal P, low complement levels, or other organs involvement should be more suggestive of SLE.

### 2.5. Behçet's Syndrome

Behçet syndrome (BS) is a systemic vasculitis characterized by mucocutaneous, ocular, and neurological involvement. Moreover a significant proportion of Behçet patients results very prone to recurrent vascular thrombosis, which could represent the main clinical manifestation of the disease, so complicating the differential diagnosis with APS; due to its primary inflammatory vascular origin, the thrombotic events in BS need corticosteroid or immunosuppressive therapy rather than anticoagulation [16].

Noteworthily the inappropriate use of anticoagulants in BS could be harmful if used in patients with clinically occult pulmonary artery aneurisms, since the risk of rupture is very high in this setting [17].

Of note as APS also BS can be responsible for cerebral venous sinus thrombosis and parenchymal lesions [18, 19]. Neuro-Behçet (NB) brain inflammatory lesions are typically located deeply at the level of basal ganglia and brainstem, while APS usually involves the subcortical and periventricular white matter areas; this differential diagnosis is decisive because, even in this case, NB requires immunosuppressive treatment [20].

In summary aPL can be found in Behçet patients with doubtful clinical significance [21]. However, recurrent bipolar aphthosis and ocular involvement, when present, are highly specific for BS.

## 3. Definite APS with Unusual Clinical Manifestations

Thrombotic manifestations of APS may have an unclear clinical presentation when they occur in atypical sites.

### 3.1. Neurological Involvement

Cerebral ischemic events are the main neurological manifestations of APS, while cerebral venous thrombosis is considered rare events but must always be considered in diagnostic workup because signs and symptoms may be unclear and diagnosis may be difficult without a specific approach [22].

### 3.2. Renal Involvement

Kidney manifestations are less frequent and less recognized in APS than in SLE patients and mainly differ for their primary vascular involvement with only secondary glomerular damage [23, 24]. Indeed the main events are renal vein thrombosis, characterized by sudden clinical presentation as untreatable hypertension, secondary nephrotic syndrome, and renal infarction. Another manifestation is kidney microangiopathy affecting afferent arterioles and clinically characterized by hypertension and laboratory signs of renal failure. Histological alterations are marked by thrombotic microangiopathy with fibrin deposition and microthrombi causing secondary glomerular dysfunction, interstitial fibrosis, and tubular atrophy; the presence of aPL and typical histopathologic features, involving both arterioles and glomerular capillaries, are the hallmarks of APS associated nephropathy [25, 26]. In clinical practice, for patients with SLE and aPL positivity, a biopsy procedure is necessary to differentiate inflammatory damage from thrombotic damage, since the therapeutic approaches are quite different; moreover, renal damage due to APS on kidney histology seriously affects lupus nephritis outcome and long-term anticoagulant therapy has been recommended for such patients [27].

### 3.3. Gastrointestinal Involvement

Thromboses of arterial or venous districts such as sovraepatic (Budd-Chiari syndrome), portal, mesenteric and more rarely of the splenic veins are the main events of gastrointestinal involvement in APS patients; differential diagnosis in these cases should be myeloproliferative disorders and in such cases the determination of JAK2 is crucial [28]. Henoch-Schönlein purpura and polyarteritis nodosa should be considered in differential diagnosis with APS with prevalent involvement of gastrointestinal tract, even though, differently from vasculitides, APS usually is not associated with increased serum levels of inflammatory markers. Noteworthily gastrointestinal events are more frequent in CAPS which should be always suspected in patients who present with rapid and severe multiorgan ischemic dysfunction [29].

### 3.4. Endocrinological Involvement

Adrenal insufficiency secondary to acute vascular infarction is the main, even though rare, manifestation of APS but diagnosis can be hard [30].

## 4. Incomplete Pregnancy Manifestations

Pregnancy disorders not completely fulfilling Miyakis criteria (i.e., 2 consecutive abortions or 3 or more nonconsecutive abortions before the 10th week of gestation) may raise the suspicion of APS; however, many other clinical conditions must be excluded, such as anatomic dysfunction, endocrine disorders, coagulopathies, or other autoimmune diseases, for example, SLE, autoimmune thyroiditis, or celiac disease [31].

## 5. Noncriterial Clinical Clues

Differential diagnosis results more complex for such noncriterial clinical clues whose presence does not allow, according to Miyakis consensus, a diagnosis of definite APS, even though this clinical presentation strongly suggests the suspicion of APS or the potential evolution over time towards it. These patients represent a significant proportion of subjects to be prospectively evaluated in order to detect early manifestations of definite disease.

### 5.1. Neurological Involvement

Brain MR imaging could be similar for morphology and location in multiple sclerosis (MS) and APS with SNC involvement [32]; moreover, ANA and aPL (especially anti-*β*2GPI of IgM isotype) could be present also in MS patients, making in some cases the differential diagnosis between the two conditions more difficult. Usually the diagnosis of demyelinating disease is supported by the presence of lesions involving the periventricular and corpus callosumareas and the detection of oligoclonal bands from cephalospinal fluid [33, 34].

Devic's syndrome, nowadays known as neuromyelitis optica (NMO), is considered a distinct disorder from MS, since it recognizes a different inflammatory pathway and the presence of aquaporin-4 (anti-NMO) antibodies is considered the main pathogenetic player and a diagnostic biomarker. In particular testing patients for anti-NMO antibodies is strongly recommended in subjects who present severe optic neuritis (usually more serious than in MS) and/or with transverse myelitis longitudinally extended, defined as a spinal inflammatory lesion involving at least three consecutive vertebrae [35].

NMO can coexist with SLE and APS in the same patient; diagnose NMO typical ocular/spinal pattern investigating the presence of anti-NMO is crucial, since this lead to the correct treatment with plasmapheresis or other immunosuppressive treatment [36].

### 5.2. Cutaneous Involvement

Livedo, acrocyanosis, and peripheral ulcers represent the most likely cutaneous manifestations of APS [37, 38].

Livedo of the limbs, the most characteristic cutaneous manifestation in APS patients, is histologically marked by partial or complete lumen occlusion of small or medium caliber arteries of dermo-hypodermis without perivascular inflammatory infiltrates. Of note only livedo racemosa (irregular and interrupted borders) is associated with pathological conditions such as APS, while livedo reticularis (circular and continuous borders) is considered a benign condition and is more commonly encountered during clinical practice [39].

A debate exists about differential diagnosis between APS and Sneddon's syndrome, since both are characterized by the presence of cerebrovascular accidents and cutaneous manifestations such as peripheral ulcers and livedo. Markers for differential diagnosis could be considered in Sneddon's syndrome patients as follows: the presence of high blood pressure, the extent of livedo (generally very pronounced), and the absence of circulating aPL. Skin biopsy can show in selected circumstances histological findings of endothelial cell proliferation and occlusion of the small cutaneous vessels, more related to Sneddon's syndrome [40].

### 5.3. Nonthrombotic Cardiac and Pulmonary Involvement

Heart valves dysfunctions, generally of the mitral valve, ranging from mild valve thickening to the typical nonbacterial thrombotic lesions (Libman-Sacks endocarditis) have been demonstrated by echocardiographic studies [41]. Cardiomyopathy in APS patients is of a quite rare occurrence and a convincing pathogenetic relationship with circulating aPL has not been clearly demonstrated [42].

The main pulmonary nonthrombotic manifestations in APS patients are considered as follows: intra-alveolar hemorrhage, acute respiratory distress syndrome (ARDS), and fibrosing alveolitis [43, 44].

Of note, aPL are often associated with chronic thromboembolic pulmonary hypertension [45].

### 5.4. Ocular Involvement

Amaurosis fugax is the most frequent manifestation, generally without pathological fundoscopic findings, and it may represent, rather than an ocular dysfunction, a warning bell of cerebrovascular disease (i.e., transient ischemic attack). On the other hand severe ocular events which may occur in APS patients are artery or vein thrombosis, retinal vascular occlusive retinopathy, and ischemic optic neuropathy, although none of them is included in Miyakis criteria. Of note, other immunological conditions, such as giant cell arteritis (GCA), are able to induce ischemic optic neuropathy with rapidly progressive and severe visual impairment, but usually in APS inflammatory markers are normal. APS must be considered in differential diagnosis especially in young patients presenting with ocular vasoocclusive disease without any traditional thrombophilic risk factors [46, 47].

### 5.5. Hematological Involvement

Thrombocytopenia, previously included in classification criteria, is nowadays considered a noncriterial APS manifestation and is found in approximately 30–40% of APS patients, justifying aPL screening in every patient with idiopathic thrombocytopenia. Noteworthy is that APS-associated thrombocytopenia is generally less severe than SLE ones and rarely requires aggressive treatment [48]. In the differential diagnostic workup of APS-related thrombocytopenia one should always exclude, in addition to SLE, pseudothrombocytopenia, TTP, DIC, and HIT and, when hemolytic anemia is present, Evans syndrome, a hematological condition reported to be associated with APS [49].

### 5.6. Musculoskeletal Involvement

Articular symptoms are rare APS manifestations and, if present, could always raise the suspicion of an associated connective tissue disease. Moreover, especially in CAPS patients, aseptic bone necrosis, in particular of the femoral head, could be considered [50].

The pathogenesis of aseptic osteonecrosis is not yet definitely understood; however, it is believed that microthrombosis or vasculopathy is involved [51].

## Figures and Tables

**Table 1:** Main differential diagnosis of APS with usual clinical manifestations.

(i) Microangiopathic syndromes (TTP/HUS, HELLP)∗	
(ii) Heparin induced thrombocytopenia (HIT)	
(iii) Disseminated intravascular coagulation (DIC)	
(iv) Systemic lupus erythematosus	
(v) Behçet's syndrome	

^*^TTP: thrombotic thrombocytopenic purpura; HUS: hemolytic uremic syndrome; HELLP: hemolysis, elevated liver enzymes, and low platelets.
